# A Case of Postsurgical Necrotizing Fasciitis Invading the Rectus Abdominis Muscle and Review of the Literature

**DOI:** 10.1155/2014/479057

**Published:** 2014-02-23

**Authors:** Francesco Carbonetti, Antonio Cremona, Marco Guidi, Valentina Carusi

**Affiliations:** ^1^Department of Radiology, Sant'Andrea Hospital, Faculty of Medicine and Psychology, Sapienza University of Rome, Via di Grottarossa 1035 Cap, 00189 Rome, Italy; ^2^Department of Orthopaedics, Sant'Andrea Hospital, Faculty of Medicine and Psychology, Sapienza University of Rome, Via di Grottarossa 1035 Cap, 00189 Rome, Italy; ^3^Department of Internal Medicine, Sapienza University of Rome, Faculty of Medicine and Psychology, Via di Grottarossa 1035 Cap, 00189 Rome, Italy

## Abstract

Necrotizing fasciitis is a life-threatening, soft tissue infection and an early diagnosis is needed to permit a prompt surgical and medical intervention. Due to the high fatal potential of the disease complications, the radiologist should distinguish necrotizing fasciitis from the most common soft tissue infections, in order to permit a prompt surgical and medical treatment. We present a case of a wide necrotizing fasciitis who presented at our emergency department and we also provide the basic tools, through a review of the literature, for the general radiologist to distinguish, with computed tomography and magnetic resonance imaging, necrotizing fasciitis from the most common infections that could present during our routine practice.

## 1. Introduction 

Necrotizing infectious fasciitis (NIF) is a rare disease; incidence of necrotizing fasciitis has been reported to be between 500 and 1500 cases per year in the United States. It is a rare infection of the deeper layers of skin and subcutaneous tissues, easily spreading across the fascial plane within the subcutaneous tissue [1]. The increase in the modern society of favouring factors such as diabetes and primary and secondary immunological disorders made the disease a reality that should be known both from the radiologist and from the clinician.

## 2. Case Report

We describe a case of a 78-year-old male who 24 hours after an anterior rectum surgical resection with a terminoterminal anastomosis for a rectal neoplasm,showed sudden pain among the soft tissues of right flank and fever (39°C).

The patient did not have any primary or acquired immunodeficiency; he was under treatment for diabetes and had no other risk factors for necrotizing fasciitis such as immunological disorders or previous administration of chemotherapy.

Clinical examination showed a swollen and hot skin and “crackling snow crepitus” among the right flank. No bullae or other skin signs predicting necrosis were appreciable.

The surgeon, after the clinical examination, requested from our Emergency Department a contrast enhanced CT (CECT) of thorax and abdomen.

### 2.1. Imaging Findings

We performed a precontrast phase, arterial phase, and an early venous phase.

CT scans showed the outcomes of the surgery and two drainage pipes were present, one in the right pelvis and one in the left flank.

**Figure 1:**
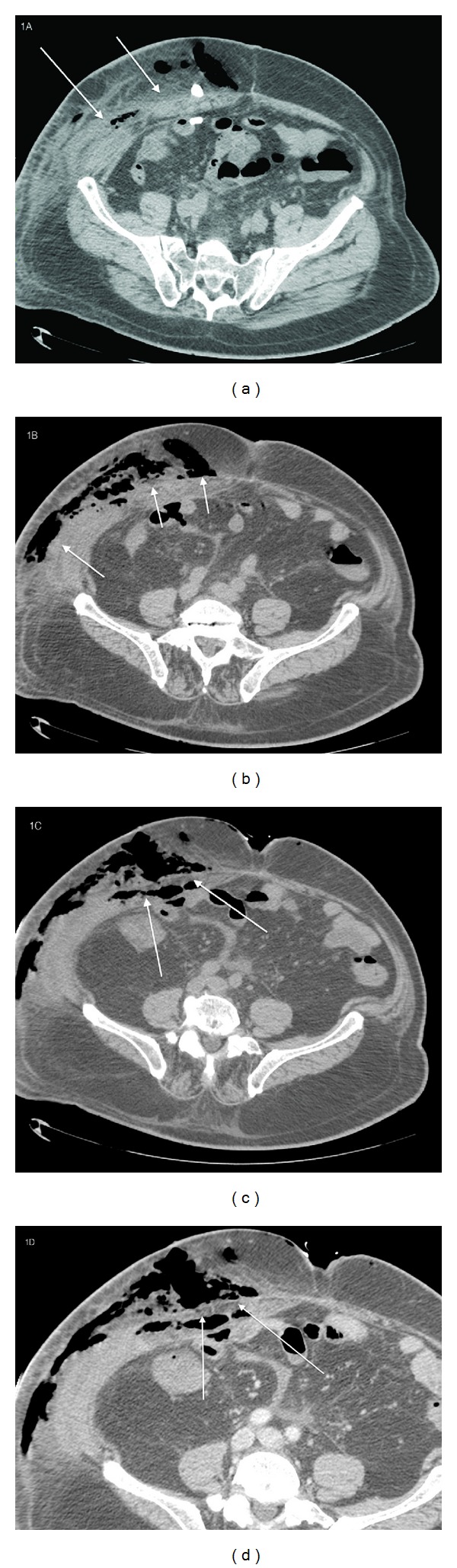
78-year-old male with diagnosis of postsurgical necrotizing fasciitis. CT scans axial ((a), (b), (c), (d)) show the presence, close to the right drainage pipe, of huge air-densities collections and edema extending along the soft tissues as right subcutaneous fat tissue, parietal abdominal muscles, and subcutaneous fascia was also involved. (a) Axial precontrast CT shows the presence of edema of the soft tissue and of the muscles which appear swollen. (b) Axial precontrast CT shows the presence of multiple huge air-densities collections among the swollen muscles. (c) CT axial precontrast. The image shows the involvement of the deep muscular fascia and the presence of air collections within it. (d) Axial precontrast CT, zoomed detail, shows the involvement of the deep muscular fascia.

Close to the right drainage access a suspected infection characterized by huge air-densities collections and edema extending along the soft tissues as right subcutaneous fat were observed; parietal abdominal muscles and subcutaneous tissues were also involved (Figure 1).

**Figure 2:**
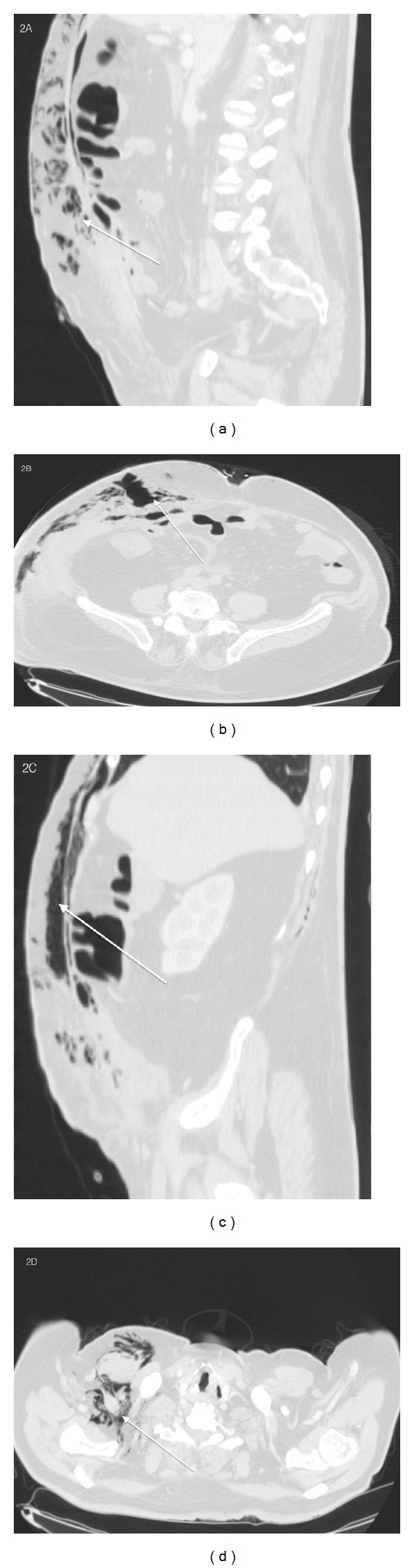
78-year-old male with diagnosis of postsurgical necrotizing fasciitis. CT axial ((b), (d)) precontrast and Sagittal Multiplanar Reconstruction ((a), (c)) show the presence of scout of subcutaneous gas, at different level, with a wide extension up to the clavicular region. (a) Precontrast Sagittal Multiplanar Reconstruction with lung window shows the presence of subcutaneous gas among the soft tissue structures and muscles. (b) Precontrast axial CT scan with lung window shows the presence of subcutaneous gas. (c) Precontrast Sagittal Multiplanar Reconstruction shows the presence of subcutaneous gas in the deeper structures of the abdominal wall and among the fat soft tissue. (d) Precontrast axial CT scan shows the extension of the subcutaneous emphysema up to the clavicular region.

**Figure 3:**
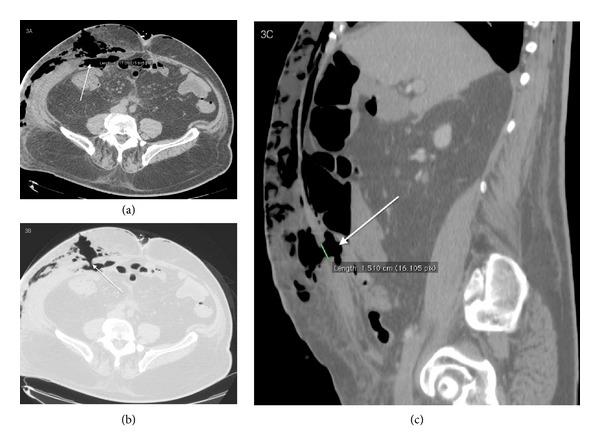
78-year-old male with diagnosis of postsurgical necrotizing fasciitis. Precontrast axial ((a), (b)) and Sagittal Multiplanar Reconstruction (c) show the presence of a solution of continuity 1,3 cm × 1,5 cm in size, of the rectus abdominis muscles. (a) Precontrast axial scan shows the presence of the solution of continuity of the rectus abdominis muscles, 1,3 cm axial diameter sized, due to the invasion of the infection of the abdominal muscles. (b) Precontrast axial scan with lung window to better identify the solution of continuity of the rectus abdominis muscles. (c) Precontrast sagittal multiplanar reconstruction shows the presence of solution of continuity of the rectus abdominis muscle sized 1,5 cm in the sagittal plane.

Scout of subcutaneous emphysema which extends up to the supraclavicular region was seen (Figure 2). Solution of continuity, 1,3 cm × 1,5 cm in size, of the rectus abdominis muscles was observed (Figure 3).

**Figure 4:**
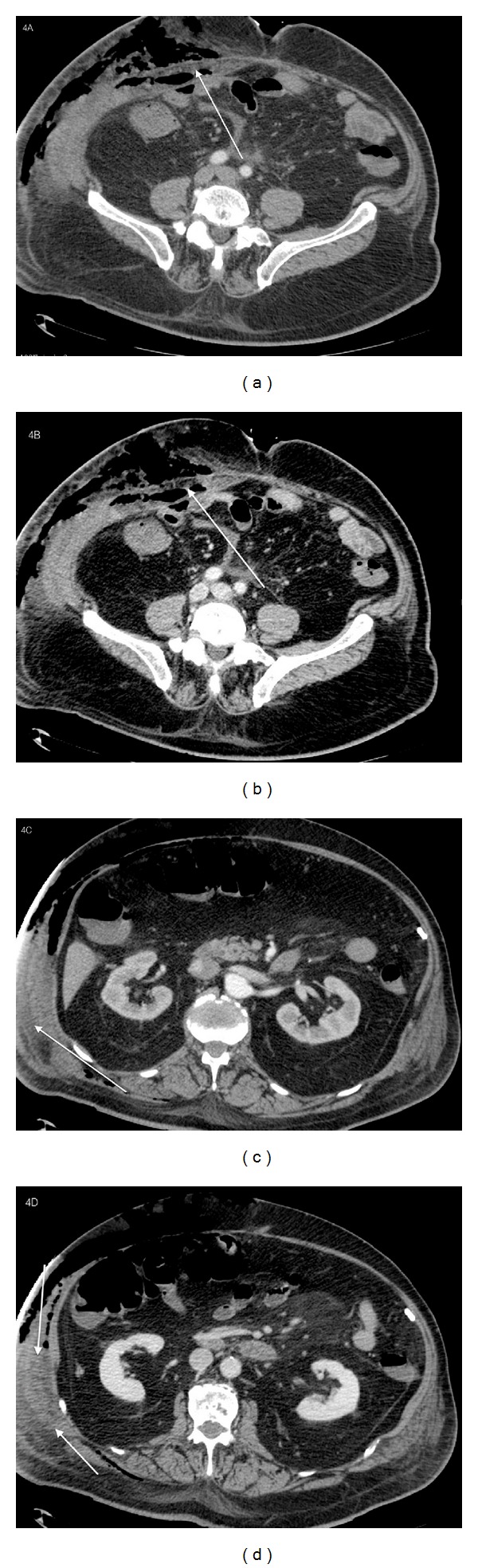
78-year old male with diagnosis of postsurgical necrotizing fasciitis. Postcontrast arterial and early venous phase, axial CT. After the administration of the contrast medium the fascia appeared as follows: not enhanced in the arterial phase (a) and weakly enhanced in the early venous phase (c), and areas of low attenuation ((c) and (d)) within the muscles and among the fat tissues structures of the right flank were observed. (a) Axial arterial phase, the fascia appeared not enhanced. (b) Axial early venous phase, the fascia appeared weakly enhanced. (c) Axial arterial phase shows the presence of areas of low attenuation among the muscles of the right flank. (d) Axial early venous phase shows the presence of areas of low attenuation among the muscles of the right flank.

After the contrast medium administration the fascia did not appear enhanced in the arterial phase; in the early portal phase the fascia appeared weakly enhanced; areas of low attenuation within the muscles and the fat tissues structures of the right flank were observed (Figure 4).

**Figure 5:**
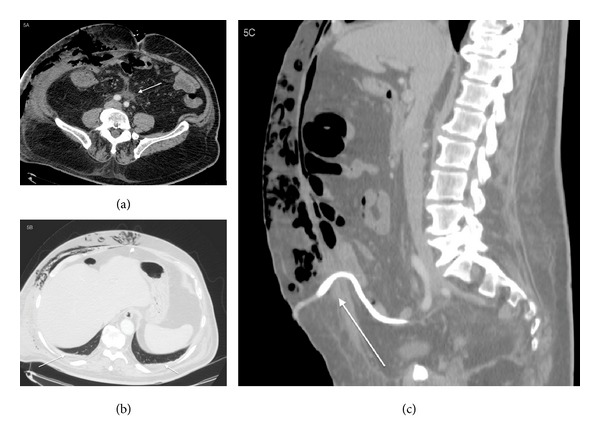
78-year-old male with diagnosis of postsurgical necrotizing fasciitis. Postcontrast arterial phase, axial CT. After the contrast medium administration the mesenteric fat tissue appeared hyperdense (a) and small amount of pleural (b) effusion was seen. These CT findings were suggestive of a systemic reaction of the patient to the musculoskeletal infection. Sagittal Multiplanar Reconstruction shows the starting point of the infection: the right drainage pipe (c). (a) Postcontrast arterial phase, axial CT. Mesenteric tissue appeared hyperdense. (b) Postcontrast arterial phase, axial CT, with lung window. Small quote of pleural effusion was seen. (c) Sagittal Multiplanar Reconstruction shows the starting point of the infection: the right drainage pipe.

As collateral findings the mesenteric fat tissue appeared hyperdense and small amount of pleural and pericardial effusion was seen; in Sagittal Multiplanar Reconstruction it was clearly appreciable that the infection started from the right drainage pipe (Figure 5).

### 2.2. Management

CT findings, such as the presence of fluid collections, the presence of subcutaneous gas, the invasion of the fascia, and the presence of areas of low attenuation within the muscles, were suggestive, in accordance with the clinical history of the patient, of a wide necrotizing infectious fasciitis (NIF) starting from the right drainage pipe with an involvement of the rectum abdominis muscles.

Signs of systemic infection, such as pleural and pericardial effusion and the hyperdensity of the mesenteric fat tissue, were also present.

Patient, due to the widespread of the disease, underwent immediately surgical debridement of the involved tissue; suture of the rectum abdominis muscles was made; intraoperative findings and the histological examination of the removed muscles confirmed the presence of necrosis among the fascia and diagnosis of NIF was made.

Antibiotics were administrated and the patient survived after the surgery.

### 2.3. Follow-Up

After the surgery clinical conditions of the patient suddenly improved; antibiotics were administrated for two weeks and no other radiological exams were performed.

## 3. Discussion

### 3.1. Etiology and Demographics

Necrotizing infectious fasciitis (NIF) is a rare disease; incidence of necrotizing fasciitis has been reported to be between 500 and 1500 cases per year in the United States [1].

NIF is a life-threatening, soft-tissue infection characterized by rapidly spreading inflammation and necrosis of the skin, involving the subcutaneous tissues and fascia due to the spread of the bacteria among and inside the skin surface. When necrosis is present, it is called NIF; otherwise in all other cases where inflammation of the deep fascia and subcutaneous tissue is present, but there was not a demonstrable necrosis, it is called non-NIF. To distinguish NIF from non-NIF is a fundamental key point due to the different treatment strategy [1].

NIF was first described by Joseph Jons, an American army surgeon during the American Civil War, and was popularized in the lay media during the 1980s after an increase in prevalence was said to be caused by “flesh-eating bacteria.” The clinical disease is expressed when infective organisms spread through the tissue along the deep fascia [2].

The disease may occur if the right set of conditions are present; these include an opening in the skin that allows bacteria to enter the body such as surgical wounds, bite of animals, scratches, burns, puncture needles, and skin open trauma.

Risk factors for the disease are diabetes, chronic disease, immunosuppressive drugs, malnutrition, age > 60 years, intravenous drug misuse, peripheral vascular disease, renal failure, primary and secondary immunological disorders, and underlying malignancy and obesity.

Microbiologically, NIF has been classified as either type I (polymicrobial), type II (monomicrobial), or type III (gas gangrene) [2].

Polymicrobial infections are more common, with cultures yielding a mixture of aerobic and anaerobic organisms. The etiologic isolates consist of Gram-positive organisms such as *Staphylococcus aureus*, *S. pyogenes*, and enterococci; Gram-negative aerobes such as *Escherichia Coli* and *Pseudomonas* species; and anaerobic organisms, such as *Bacteroides* or *Clostridium* species. Type 1 NIF occurs in immunocompromised individuals. Monomicrobial infections are less common than the polymicrobial variety.

The mortality rate may be high as 30%–70% with death being due to sepsis, respiratory failure, kidney failure, or multiorgan system failure.

Studies have shown that only 15% to 34% of patients with NIF have an accurate admitting diagnosis [3].

### 3.2. Clinical and Imaging Findings

#### 3.2.1. Clinical

Patients with NIF can present with constitutional symptoms of sepsis alone or with evidence of skin inflammation, which makes diagnosis a little more straightforward.

The most common signs of skin inflammation in NIF are skin localized pain, skin edema, and erythema with a patchy discoloration of the skin. The skin usually appeared swollen and mottled and a sense of crackling snow suggestive for the presence of subcutaneous gas could be present.

Progression of NIF is marked with the development of tense edema, a grayish-brown discharge, vesicles bullae, necrosis, and crepitus that are clear signs of the skin necrosis [2, 3].

Hemorrhagic bullae and crepitus are sinister signs, with the likelihood of underlying fascia and muscle being compromised.

The physical examination should include all parts of the body to investigate for skin inflammation.

With the spread of the disease among the tissues constitutional symptoms occur such as fever, tachycardia, altered mental state, and diabetic ketoacidosis.

*Leukocytosis* with neutrophilia, acidosis, altered coagulation profile, impaired renal function, raised creatine kinase levels, and raised inflammatory markers, such as C-reactive protein levels, are helpful if viewed within the whole of the clinical context. We should also remember that clinical scores like the indicator for NIF (LRINEC score) are available to help diagnosis of NIF and differentiate it from other skin and soft tissue infections. Blood cultures and tissue cultures should be performed [3].

#### 3.2.2. Imaging Findings

X-rays films do not play a fundamental role in the diagnosis and management of NIF because they could just reveal the presence of subcutaneous gas; thus they could be helpful in the follow-up of the patients in order to monitor the evolution of subcutaneous gas.

CT imaging is a helpful tool in distinguishing NIF from the most common infections of the soft tissues such as cellulitis, nonnecrotizing fasciitis, soft tissues abscess, infectious myositis, and osteomyelitis where there is an involvement of the bone adjacent to the infection [4].

Cellulitis is an acute infection of the dermis and of the subcutaneous tissues and usually does not involve the fascia. CT demonstrates skin thickening, septation of the subcutaneous fat, and thickening of the underlying superficial fascia. On MRI these alterations of the dermis and of subcutaneous tissues could be detected as linear streaks or as focal areas of alteration of signal, hyperintense on T2-WI and hypointense in T1-WI [5, 6]. Usually in cellulitis there is a thickening of subcutaneous tissues with fluid collections in the subcutaneous fat layer detected as areas of hyperintensity on T2-WI; the deeper fascia is not involved [5, 6].

In complicated cellulitis the deeper structures and the fascia could be involved and necrotizing fasciitis should be suspected.

The imaging findings in necrotizing fasciitis are similar to those in cellulitis but are more severe and the involvement of the fascia and of the deeper muscular structures is always present, while in cellulitis it is not a common finding.

One specific distinguishing sign of necrotizing fasciitis is the presence of gas in the subcutaneous tissues caused by gas-forming anaerobic organisms. In a study written by Yu and Habib, it is reported that the presence of gas in the subcutaneous tissues caused by gas-forming anaerobic organisms is a specific sign of NIF [7].

CT enables excellent and fast visualization of the subcutaneous gas and in the evaluation of this finding it is dramatically superior compared with MRI. On MRI gas collections result in focal foci of signal void both in T1-WI and T2-WI and it could be difficult to detect them.

Other CT findings in NIF include thickening of the affected fascia, fluid collections along the deep fascial sheaths, and extension of edema into the intramuscular septa and the muscles, findings that were present in the case we presented [8–10].

To determine if necrosis is present the administration of contrast medium could be a helpful tool that helps to distinguish the necrotic fascial tissue from the nonnecrotic one. The literature shows evidences that when necrosis is present, on CECT in the delayed phase, a lack of the enhancement of the fascia could be observed [8–10]. In our case we performed an early venous phase, so we could not express any definitive consideration about the enhancement of the fascia.

Regarding the lack of enhancement of the fascia, we have also to say that it could not be considered as a certain predictor factor of necrosis. In fact in some studies, where the efficacy of the findings predictive of NIF was studied with MRI which is more contrast sensitive to CT, a lack of enhancement of the fascia was observed not just in the patients affected by NIF but also in some patients not affected by NIF [11–13]. So the lack of enhancement of the fascia, both on CECT and on MRI, could not be considered as a certain predictor factor of necrosis but should be considered as a suggestive predictive factor of necrosis according to the other radiological and clinical findings.

Soft tissue abscess presents a well-demarcated fluid collection with a peripheral pseudocapsule showing ring enhancement on CECT and due to these characteristics it could be easily distinguished from cellulitis and fasciitis.

On MRI soft tissue abscess shows low signal intensity (SI) on T1-WI and intermediate-high SI on T2-WI, sometimes with T2 hypointense lower SI layering debris. Thick peripheral rim enhancement, with or without a central hypointensity, in a case of abscess is present after contrast medium administration on T1-WI fat sat postcontrast sequences [14]. Calcifications and gas collections could be also present in abscesses and the best technique to evaluate these two findings, as said before, is CT. MRI calcifications result in a signal loss in T2-WI and T1-WI but they are not easily and readily demonstrable as on CT. To evaluate the presence of edema, which could be present both in muscular infections and in abscesses, Short Time Inversion Recovery Sequences (STIR) should be performed. STIR sequences, which are T2-weighted sequences, reveal the presence of edema as an area with increased signal intensity compared to the normal tissues [14, 15].

When there is an infectious myositis, on CT, there are enlargement and decreased attenuation of the affected muscle with effacement of surrounding planes. Involvement of a muscle group that is disproportionate to the involvement of subcutaneous tissue helps distinguish myositis from primary cellulitis. Intramuscular fluid collections can be also present in infectious myositis; the administration of contrast medium could help to differentiate necrotic from viable musculature and to demonstrate if a rim enhancing abscess is present [10].

On MRI in case of infectious pyomyositis, hyperintense focal lesions on T2-WI with massive perifocal edema resulting in a hyperintensity on the STIR sequences could be seen [6, 7].

When there is an involvement of adjacent bone osteomyelitis can occur. A periosteal reaction, medullary low-attenuation areas, trabecular coarsening, and focal erosions are CT signs of osteomyelitis [10].

On CT, features of osteomyelitis include also soft tissue swelling, periosteal reaction, medullary low-attenuation areas or trabecular coarsening, and focal cortical erosions [10].

On MRI, in a case of acute osteomyelitis, sites of involved medullary bone appeared on T1-WI as areas of ill-defined homogenous, low to-intermediate signal intensity, and on T2-WI as areas of high signal intensity and as bright as or brighter than bone marrow. On T1-WI images there are accompanying areas of ill-defined intermediate signal intensity in the cortex and soft tissue and disruption on the normal high-intensity signal of the adjacent intramuscular septa and subcutaneous fat. These foci changed to high signal intensity on T2-WI images just as the lesions in the medullary canal had [6, 7].

Therefore the involvement and the thickening of the fascia, fluid collections, presence of subcutaneous gas, edema of the muscular septa, the involvement of the subcutaneous tissue, a nonenhancing fascia after contrast medium administration, and areas of low-attenuation in the muscles and among the soft tissues are all findings that together with the clinical history of the patient could suggest to the radiologist the presence of NIF [4, 8–10]. Other signs of systemic infection, such as pleural effusion or the hyperdensity of the fat mesenteric tissue, as seen in our case could be present in NIF when a systemic reaction is present [4, 8–10].

In case of NIF, MRI imaging could be a useful tool in the evaluation of the precise extent of the infection and of its relations with the surrounding organs, to distinguish mild fascial or muscle involvement and to determine, with a higher sensibility compared to the CT scan, if necrosis and edema are present or not. Generally fat-suppressed T2-WI has been found to display inflammatory changes better than fat-suppressed gadolinium-enhanced T1-WI [11, 12]. On MRI scan patients with NIF usually present thick (>3 mm) areas with abnormal high signal intensity on fat-suppressed T2-WI in the deep fascia, with a less frequency combined areas in superficial and deep fascia of low signal intensity on fat-sat T2-WI; a focal or diffuse nonenhancing portion in the area of the high abnormal signal intensity, in the deep fascia, in T1-WI fat sat postcontrast images [11–13]. Regarding the presence of a focal or diffuse nonenhancing portion after contrast medium administration on T1-WI fat-sat postcontrast images, it applies the considerations said above in the text.

MRI is a second stage exam that could be performed in all those doubt cases, after a first CECT evaluation, to distinguish NIF from non-NIF. MRI is dramatically inferior compared to CT in the evaluation of the presence of subcutaneous gas and calcifications. MRI is more contrast sensitive compared to CT; it permits a better evaluation of the precise extent of the disease and it offers an important diagnostic adjunct to the management of NIF and the potential to defer or limit extensive exposure of the deep fascia [11–13]. Nuclear imaging studies (99 mTC-Phosphate Complex and 67 Ga) are useful in those cases where a complication of osteomyelitis is suspected [16, 17].

### 3.3. Treatment and Prognosis

Once the diagnosis is made, treatment must begin on multiple fronts.

First, surgical consultation should be urgently requested with the intention of early wound debridement for collection of tissue cultures, excision of all nonviable and necrotic tissues should be done, and delineation of the extent of the disease tissues should be done. If the necrosis is present or strongly suspected, both with clinical examination and with the radiological findings, surgical debridement is mandatory.

In NIF a surgical debridement is mandatory; nonnecrotizing fasciitis does not require emergency surgery but affected patients should be monitored because of the potential necrosis.

Until blood culture results are available, wide spectrum antibiotic coverage with intravenous administration should be started.

The antibiotic should cover *pyogenes*, *aureus,* and Gram-negative aerobes and anaerobes as clinically indicated.

Hyperbaric oxygen has also been used as an adjunct to surgery and antibiotics.

Mortality due to NIF is considerable. Without surgical interventions, mortality approaches 100% [18]. More recent data indicate a mortality of 16,4% for community-acquired necrotizing soft tissue infections and 36,3% for postprocedural necrotizing fasciitis infections. Mortality is higher in patients with streptococcal toxic shock syndrome and in diabetic patients.

A delay to surgery of more than 24 hours was an independent risk factor for mortality. There is also considerable postoperative morbidity, sometimes from extensive debridement resulting in muscle or limb loss [19].

## 4. Conclusion

In conclusion, even if the diagnosis of NIF is both clinical and radiological, in the literature there are specific CT and MRI signs that allow the radiologist to distinguish NIF from non-NIF and from the most common musculoskeletal infections in order to perform a prompt surgical intervention when NIF is suspected.

Both techniques, CT and MRI, as said before have their weak and strength points, and as a general consideration we can say that CECT should be performed as first stage exam following with MRI in the doubt cases.

Some predisposing factors such as diabetes, previous surgery, or wounds in the skins should alert the radiologist of the possible presence of deep muscular infections.

The main goal for the radiologist at the emergency department when a deep muscular infection is suspected is to distinguish NIF from nonnecrotizing fasciitis and from other musculoskeletal infections and to evaluate the extension of the disease, the presence of necrosis if it is possible, and the disease complications both local and systemic.

## 5. Differential Diagnoses

Nonnecrotizing fasciitis, infectious myositis, cellulitis, soft tissues abscess, and osteomyelitis.

## Abbreviations

NIF: Necrotizing infectious fasciitis
CECT: Contrast enhanced CT
MRI: Magnetic resonance imaging
T1 and T2 WI: T1-weighted images, T2-weighted images
STIR: Spin echo time inversion recovery
SI: Signal intensity.
